# Psychometric Properties of the Nine-Item Problematic Internet Use Questionnaire in a Brazilian General Population Sample

**DOI:** 10.3389/fpsyt.2021.660186

**Published:** 2021-05-12

**Authors:** Daniel Tornaim Spritzer, Wagner de Lara Machado, Marina Balem Yates, Vitória Rech Astolfi, Pricilla Laskoski, Cristina Pessi, Stéphanie Laconi, Katarzyna Kaliszewska-Czeremska, Zsolt Demetrovics, Orsolya Király, Ives Cavalcante Passos, Simone Hauck

**Affiliations:** ^1^Graduate Program in Psychiatry and Behavioral Sciences, Universidade Federal do Rio Grande do Sul, Porto Alegre, Brazil; ^2^Graduate Program in Psychology, Pontificia Universidade Catolica do Rio Grande do Sul, Porto Alegre, Brazil; ^3^Centre d'Études et de Recherche en Psychopathologie et Psychologie de la Santé, Université Toulouse Jean Jaurès, Toulouse, France; ^4^Institute of Psychology, Jesuit University Ignatianum in Krakow, Krakow, Poland; ^5^Centre of Excellence in Responsible Gaming, University of Gibraltar, Gibraltar, Gibraltar; ^6^Institute of Psychology, ELTE Eötvös Loránd University, Budapest, Hungary; ^7^Laboratory of Molecular Psychiatry, Centro de Pesquisa Experimental and Centro de Pesquisa Clínica, Hospital de Clínicas de Porto Alegre, Porto Alegre, Brazil; ^8^Psychodynamic Psychiatry Research Laboratory, Hospital de Clínicas de Porto Alegre, Porto Alegre, Brazil

**Keywords:** internet addiction, Problematic Internet Use Questionnaire, cultural adaptation, psychometrics, Brazil

## Abstract

**Objective:** The goal of the study is to adapt and examine the psychometric properties of the Brazilian version of the nine-item Problematic Internet Use Questionnaire (PIUQ-SF-9).

**Methods:** A convenience sample of Brazilian internet users aged between 18 and 89 years (72.7% female, mean age 38.7 years ± 13.5) was recruited online from September 2018 to July 2019 (test sample = 1,525; retest sample = 237). Participants responded to the adapted version of the PIUQ-SF-9, as well as the Center for Epidemiologic Studies-Depression Scale (CES-D-10) and sociodemographic questions.

**Results:** A bifactor model with one general factor and three specific dimensions (obsession, neglect and control disorder) yielded the best fit indices [χ^2^ = 67.66, df = 15, CFI = 0.99, TLI = 0.99, RMSEA = 0.048 (0.037–0.060), RMSEA *p* close = 0.587 and SRMR = 0.01]. McDonald's hierarchical omega coefficient was 0.76 for the general factor and varied between 0.16 and 0.33 for the specific dimensions. The intraclass correlation coefficient was 0.73 for the general factor and varied between 0.64 and 0.72 for the specific dimensions. The MIMIC model supported the scale's construct validity as the relationship of the predictors (age, time spent online, self-perception of problematic internet use, and depression symptoms) with the PIUQ-SF-9 factors was in line with the assumptions based on the literature.

**Conclusion:** PIUQ-SF-9 seems to be a brief and culturally validated instrument with sound psychometric properties to be used in future studies on problematic internet use in the Brazilian population.

## Introduction

The internet has become an integral part of most people's lives, and in some cases, its countless benefits seem to give way to negative consequences from its overuse. Problematic internet use can be defined as excessive and uncontrolled internet use associated with significant impairment in the individual's physical and emotional health, social relationships, and professional life (1, 2). Its occurrence is associated with high rates of psychiatric comorbidities, such as depressive and anxiety disorders (3), and it is more prevalent in adolescents and young adults, who use the internet for longer periods of time than other age groups (4). Problematic internet use is an umbrella term that includes behaviors related to gaming, social network use, and access to pornography, among others (5).

In recent years, Brazil has undergone a major technological revolution, and it is estimated that ~75% of the Brazilian population has regular access to the internet, which corresponds to more than 150 million individuals (6). Research on the problematic internet use in Brazil is on the rise, as it is in many countries. However, one of the existing difficulties is the paucity of instruments available to study this phenomenon, since only the Internet Addiction Test [IAT; (7, 8)], and the Online Cognition Scale [OCS; (9–11)] have been translated and culturally adapted to Brazilian Portuguese. The IAT, despite being the most widely used, had no psychometric properties other than the internal consistency assessed in our population. The OCS has been assessed for semantic equivalence, reliability, and construct validity among university students, but it has been considerably less investigated, perhaps because it is longer and more time-consuming. The development of measurement tools that are valid, reliable, and validated across cultural settings is essential for screening people in risk of problematic internet use, investigating clinical and etiological aspects of this phenomenon, and evaluating prevention and treatment strategies (2).

The Problematic Internet Use Questionnaire—Short Form-9 (PIUQ-SF-9) is a nine-item comprehensive screening tool assessing three basic aspects of problematic internet use: obsession (i.e., preoccupation and withdrawal symptoms), neglect (i.e., negligence of everyday activities and basic needs), and control disorder (i.e., trouble in controlling internet use) (12). Several studies have examined its psychometric properties, showing high internal consistency, replicable factor structure, and moderate to good test–retest properties. It has also proved to be valid across various methods of data collection (i.e., online as well as paper–pencil) and age groups, being considered suitable for time-limited surveys (12, 13). Cross-cultural psychometric studies found that the PIUQ-SF-9 demonstrated adequate measurement invariance across several European and Asian countries (14–16).

The aim of the present study is to describe the cultural adaptation process of the PIUQ-SF-9 to Brazilian Portuguese and the evaluation of its psychometric properties in a general population sample.

## Materials and Methods

### Cultural Adaptation Process

The original instrument (including instructions, all items and answer possibilities) was independently translated by six bilingual translators whose native language was Brazilian Portuguese, divided into two groups (two psychologists and one psychiatrist in each group). The translated versions were examined by an expert committee to assess semantic discrepancies (including linguistic and conceptual issues), and, by consensus, a synthesized version of the translation was developed. The expert group was composed of 15 members skilled in psychometric research and also in internet use disorders.

It was then back translated into English by two native English speakers who worked independently to produce back-translations. The first back-translator was a psychologist born in the USA, and the second was an English teacher born in England, and both of them have lived in Brazil for many years. None of the translators previously knew the questionnaire being adapted, and they were not informed about the objectives of the study. The back-translated versions were then evaluated by two independent groups, composed of three members each, to verify how much the instructions, each item and the answer possibilities differed from the original instrument in relation to their meaning, rating on a four-point Likert scale from 1 (much altered) to 4 (not altered). At a new meeting of the experts' group, based on the insights from the back-translations' evaluations, all items on the scale were revised and, when necessary, consensually adjusted to maintain the meaning of the original instrument, producing a new synthesized and unified version in Brazilian Portuguese. A synthesized version of the back-translation was also produced and, along with the description of the adaptation process, were forwarded to the PIUQ-SF-9 authors for appraisal.

This version was then sent, in an online format, to a group of 15 people to investigate the face validity of the instrument, that is, whether the items, instructions and response scale were comprehensible to the target population (17). Comments and suggestions regarding clarity and comprehensibility were requested for each item, as well as for the whole questionnaire.

### Sample and Procedures

A convenience sample of Brazilian internet users aged between 18 and 89 years was recruited online *via* social media platforms and email, between September 2018 and July 2019. Data collection was carried out anonymously through the online research platform Survey Monkey, and the questionnaire could be accessed and answered *via* smartphone, computer, or tablet. At the end of the questionnaire, participants were offered feedback on their results from the questionnaire on problematic internet use, for which an email address was requested. Those who provided the email address were invited, in August 2019, to answer the PIUQ-SF-9 scale again for the retest validation. The invitation was made in an automated way so that the researchers did not have contact with the participants' email contacts. The time between the test and the retest was at least 4 weeks.

All participants who filled in the sociodemographic data and completed at least 90% of the PIUQ-SF-9 were included. The missings were at random and were excluded from subsequent analyzes. The sample size estimate was 1,000, considered “excellent” for carrying out the confirmatory factor analysis (CFA) and other psychometric testings (18, 19).

This cross-sectional study is part of a multicentric project carried out in 16 countries, whose main objective is to assess cross-cultural aspects of internet and smartphone problematic use. The study was approved by the Research Ethics Committee of the Hospital de Clínicas de Porto Alegre (protocol number 89702318.2.0000.5327), and it was conducted in accordance with the Declaration of Helsinki.

### Variables and Measures

Sociodemographic and internet use data: participants were asked about their age, sex, education, working and marital status, as well as number of hours of daily internet use, main device for internet connection, and self-perception of problematic internet use.

PIUQ-SF-9, (12): this questionnaire consists of nine items, which evaluate problematic internet use based on the three-factor structure of the original 18-item instrument (20): obsession, neglect, and control disorder. All questions have five-point Likert-type answers, ranging from 1 “never” to 5 “almost always/always.” Total scores range from 9 to 45, and higher scores indicate a higher risk of problematic use. Internal consistency, measured by Cronbach's alpha, varies between 0.91 and 0.93 for the whole instrument and between 0.77 and 0.89 for specific dimensions (12, 21). Test–retest reliability varies between 0.61 and 0.90 for the whole instrument and between 0.53 and 0.90 for the specific factors (20, 21).

Center for Epidemiologic Studies—Depression Scal-10 [CES-D-10; (22–24)]: it is a brief version of the CES-D, which aims to assess depressive symptoms. It consists of 10 items that are evaluated on a Likert-type scale ranging from 0 (“rarely or never”) to 3 (“most of the time or all the time”). Scores can range from 0 to 30 and, according to Andresen et al. (22), scores ≥ 10 suggest significant depressive symptomatology. In the original study and also in recent validations, Cronbach's alpha is higher than 0.80 in all subgroups (23, 25).

### Statistical Analysis

The analyses were performed in R environment (version 3.2.2) implemented by the *lavaan* package (26). In addition, *semTools* package was used to estimate reliability measures (27), and *semPlot* package was used to produce the MIMIC diagram (28). For ordered categorical variables, the Diagonal Weighted Least Squares estimation method and polychoric correlation coefficients were used with robust estimation of the means, variances, and standard errors.

CFA was performed to verify the structural validity of the instrument. In addition to the analysis of the fitted model suggested by the original study (12), other three alternative models were also evaluated as proposed by a recent study that verified the psychometric properties of this questionnaire in nine European countries (14). The model originally proposed is composed of three oblique factors (neglect, obsession, and control disorder). The alternative models presented are: (1) two oblique factors model in which the dimensions neglect and control disorder belong to the same factor; (2) bifactorial model composed of a general factor and three specific dimensions (neglect, obsession, and control disorder); and (3) bifactorial model consisting of the general factor and two specific dimensions, in which neglect and control disorder compose the same dimension. The fit indices considered to compare the model's adequacy were: Comparative Fit Index and Tucker-Lewis Index (CFI and TLI, ≥0.95), Root Mean Square Error of Approximation (RMSEA, ≤0.06) with associated *p*-value and Standardized Root Mean Residual (≤0.10) (29).

The internal consistency was assessed using McDonald's hierarchical omega coefficient (ωH), considering satisfactory if higher than 0.70 (but in the case of a bifactor model, this parameter is valid only for the general factor, once the specific dimensions scores are controlled for the variance due to the general factor) (30). Cronbach's alpha (α) was also reported for the sake of comparability with previous research. To estimate test–retest reliability, the intraclass correlation coefficient (ICC) and corresponding 95% confidence interval (CI) were calculated, and reliability was considered adequate for values between 0.50 and 0.75, good for values between 0.75 and 0.90, and excellent for values > 0.90 (31).

We also conducted a Multiple Indicators Multiple Causes (MIMIC) model to explore construct validity by estimating simultaneously the influence of possible predictors (age, time spent online, self-perception of problematic use, and depression symptoms) on the PIUQ-SF-9 general and specific factors, *via* standardized partial regression coefficients. The MIMIC is a variety of Structural Equation Modeling, which describes the effects of covariates on latent variables and the inter-relationships of latent variables, thus providing better insight than traditional correlational analysis (32, 33). Based on the literature, it was assumed that age would have a negative effect on the PIUQ-SF-9 factors while time spent online, self-perception of problematic internet use, and depression symptoms would have a positive effect on the PIUQ-SF-9 factors.

Floor and ceiling effects are considered to be present if more than 15% of respondents achieved the lowest or highest possible score, respectively (34). The dataset and the analysis script were uploaded can be accessed from the following link: https://github.com/wagnerLM/PIUQ/blob/main/PIUQ-SF-9_script_data.R.

## Results

### Cultural Adaptation

The two forward translations achieved very similar results, and only minor adjustments were needed to obtain by consensus, a synthesized version. All back-translation items were considered to be unaltered from the original instrument in relation to their meanings. No adjustment needs were identified by the authors of the original instrument when evaluating the synthesis of the back-translations. On pre-test, all respondents rated the questionnaire as “easy to understand,” and there were only a couple of minor suggestions involving word order and replacement of a term by a synonym. The final Brazilian Portuguese version of the PIUQ-SF-9, as well as the original English version, are available in Appendices A, B, respectively.

### Demographic Data

A total of 1,525 people answered the sociodemographic part, the questions about internet use and the PIUQ-SF-9 (72.7% were female, mean age was 38.7 years ± 13.5). A total of 735 participants informed their email at the end of the questionnaire and were invited to complete the PIUQ-SF-9 retest. Thus, 237 out of the 735 responded to the PIUQ-9 retest (74.7% were female, mean age was 38.1 years ± 13.8), in an average time of 6 months after the original completion. The main sociodemographic data of the test and retest samples are presented in Table 1.

**Table 1:** Descriptive statistics of sociodemographic variables.

	**Test sample**	**Retest sample**
	**(*n* = 1,525)**	**(*n* = 237)**
Mean age in years (SD)	38.75 (13.55)	38.08 (13.80)
**Gender**		
Women (%)	1.106 (72.7%)	177 (74.7%)
**Occupation (%)**		
Studying only	217 (14.2%)	41 (17.3%)
Studying and working	368 (24.2%)	66 (27.8%)
Working only	817 (53.6%)	105 (44.3%)
Not working, not studying	121 (7.9%)	25 (10.5%)
**Educational level (%)**		
Elementary School	15 (1.0%)	–
High school, incomplete	17 (1.1%)	–
High school, complete	74 (4.9%)	8 (3.4%)
High school, complete + 1–3 years of study	163 (10.7%)	33 (14.1%)
High school, complete + 4–6 years of study	311 (20.3%)	43 (18.4%)
High school, complete + 7 or more years of study	931 (61.1%)	150 (64.4%)
**Marital status (%)**		
Single	389 (25.6%)	73 (30.8%)
Dating	191 (12.6%)	34 (14.3%)
Living together	238 (15.6%)	28 (11.9%)
Married	566 (37.2%)	80 (33.8%)
Divorced	118 (7.8%)	18 (7.6%)
Widowed	18 (1.2%)	4 (1.7%)

*SD, Standard deviation*.

### Psychometric Properties

We evaluated the factor structure of the PIUQ-SF-9 testing the four models previously proposed by Laconi et al. (14) and the fit indices are reported in Table 2. The bifactor model with one general factor and three specific dimensions (obsession, neglect, and control disorder) yielded the best fit to the data [χ^2^ = 67.661, df = 15, CFI = 0.99, TLI = 0.99, RMSEA = 0.048 (0.037–0.060), RMSEA *p* close = 0.587 and SRMR = 0.01]. Factor loadings for this model are presented in Table 3. After confirming the factorial structure of the instrument, a normative table was produced, which can be found in Appendix C.

**Table 2:** Confirmatory factor analysis of four measurement models of the PIUQ-SF-9.

	**χ**^**2**^	**df**	***p***	**CFI**	**TLI**	**RMSEA (95% CI)**	**RMSEA *p*-close**	**SRMR**
Three-factor model	528.277	24	<0.001	0.97	0.96	0.117 (0.109–0.126)	0.000	0.05
Two-factor model	668.107	26	<0.001	0.97	0.95	0.127 (0.119–0.136)	0.000	0.06
Bifactor model with three specific dimensions	67.661	15	<0.001	0.99	0.99	0.048 (0.037–0.060)	0.587	0.01
Bifactor model with two specific dimensions	218.172	17	<0.001	0.99	0.98	0.088 (0.078–0.099)	0.000	0.03

*PIUQ-SF-9, Problematic Internet Use Questionnaire–Short Form−9 items; χ^2^, chi-square; df, degrees of freedom; CFI, comparative fit index; TLI, Tucker-Lewis Index; RMSEA, root-mean-square error of approximation with 95% confidence interval; SRMR, standardized root mean residual*.

**Table 3:** Standardized factor loadings and reliability indicators of the bifactor model with three specific dimensions of the PIUQ-SF-9.

	**General factor**	**Specific dimensions**		
		**Neglect**	**Obsession**	**Control disorder**
Item 1	0.64	0.47		
Item 3	0.57	0.49		
Item 9	0.70	−0.01		
Item 5	0.72		0.48	
Item 7	0.56		0.63	
Item 8	0.69		0.52	
Item 2	0.72			0.53
Item 4	0.75			0.56
Item 6	0.87			−0.08
ωH	0.76	0.16	0.33	0.18
α	0.91	0.73	0.88	0.86
ICC	0.73	0.72	0.68	0.64
ICC 95% CI	0.67–0.79	0.66–0.78	0.61–0.75	0.56–0.71

*PIUQ-SF-9, Problematic Internet Use Questionnaire – Short Form – 9; ωH, McDonald hierarchical omega coefficient; α, Cronbach's alpha; ICC, Intraclass correlation coefficient; ICC 95% CI, 95% confidence interval*.

*The significance test showed p < 0.001 for all intraclass correlation coefficients*.

Regarding internal consistency of PIUQ-SF-9, ωH was 0.76 for the general factor and varied between 0.16 and 0.33 for the specific dimensions. Cronbach's alpha was 0.91 for the general factor and varied between 0.73 and 0.88 for the specific dimensions. For the test–retest reliability, ICC was 0.73 for the general factor and varied between 0.64 and 0.72 for the specific dimensions (complete results for both internal consistency and test–retest reliability measures are presented in Table 3).

**Figure 1:**
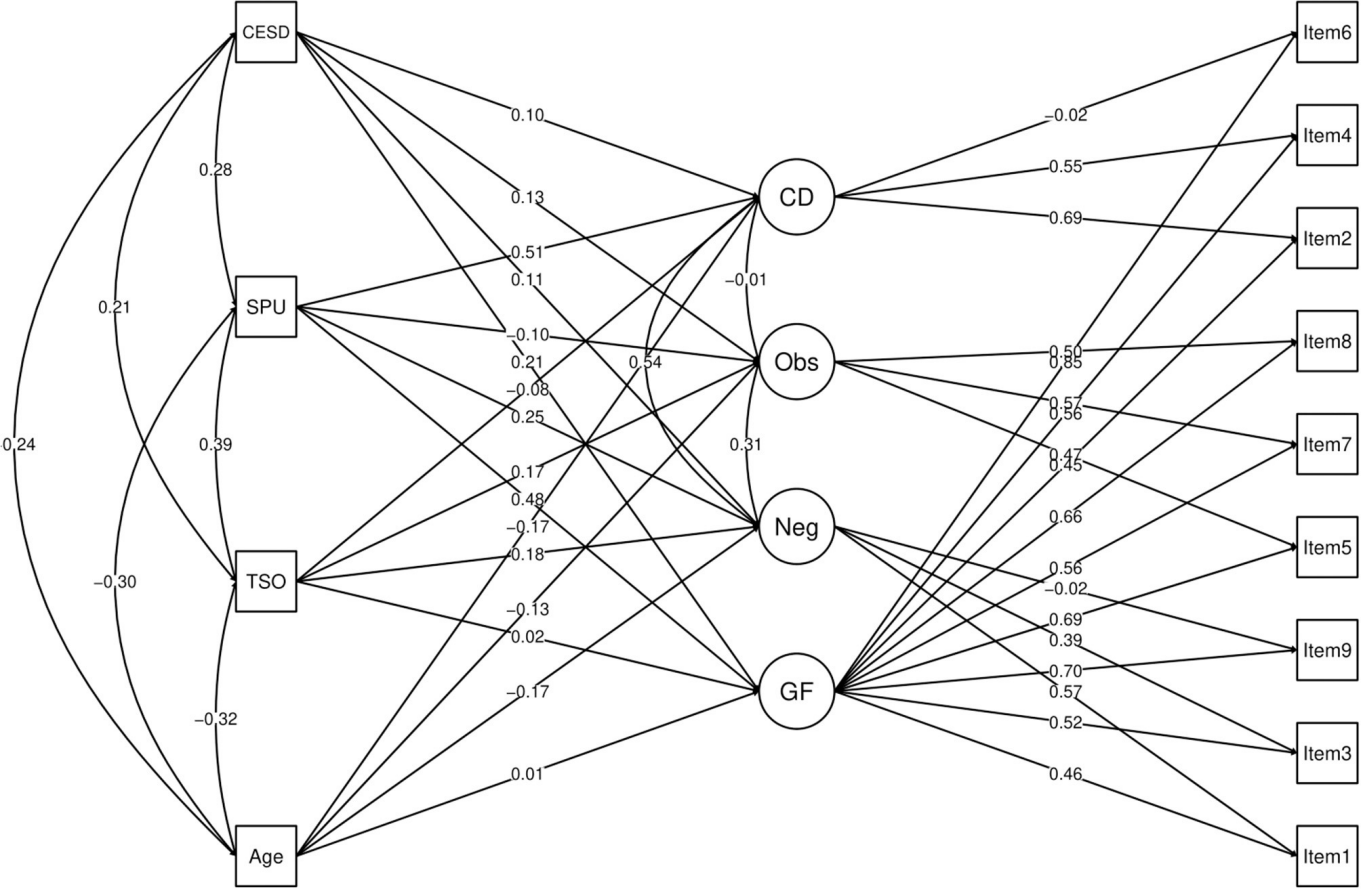
Diagram of the MIMIC model of the PIUQ-SF-9 general factor and specific dimensions and the age of the respondents, time spent online, self-perception of problematic internet use, and depressive symptoms. MIMIC, Multiple Indicators Multiple Causes; CESD, Center for Epidemiologic Studies-Depression Scale; SPU, Self-perception of Problematic Use; TSO, Time Spent Online; CD, Control Disorder; Obs, Obsession; Neg, Neglect; GF, General Factor. A detailed measurement model assessment as Supplementary Material can be found on the following link: https://github.com/wagnerLM/PIUQ/blob/main/PIUQ-SF-9_fit.

The MIMIC model had an excellent fit to the data [χ^2^(35) = 108.9, CFI/TLI = 0.99, RMSEA = 0.04 (90% C.I. = 0.03–0.05)]. Predictors explained the variance of the general and specific factors as follows: general factor = 34%, neglect = 25%, obsession = 8%, and control disorder = 35%. According to the model, the age of the respondents had no effect on the general factor, but a small negative effect on all the specific factors. Time spent online had a small positive effect on neglect and obsession, but no effect on the general factor and control disorder. Self-perception of problematic internet use had a large positive effect on general factor and control disorder, a small effect on negligence, and no effect on obsession. Depression symptoms had a small positive effect on the PIUQ-SF-9's general factor and all specific dimensions. A diagram of PIUQ-SF-9's factorial structure and the results of all regressions paths and correlations, as well as a Supplementary Material link for the detailed measurement model assessment, are presented in Figure 1.

Regarding floor and ceiling effects, 4.2% of the sample answered the minimum value for PIUQ-SF-9, while 0.1% answered the maximum value, which were considered satisfactory.

## Discussion

This study aimed at culturally adapting the PIUQ-SF-9 for use in Brazil, as well as examining its psychometric properties. Our findings demonstrated that the questionnaire has shown the best fit in the bifactor model (one general factor and three specific dimensions: neglect, obsession, and control disorder). Therefore, reliability and validity tests were carried out taking into account this factorial structure. The Brazilian Portuguese version of the PIUQ-SF-9 has shown good internal consistency and the test–retest procedures highlighted moderate stability. Construct validity was demonstrated with the MIMIC model, by the means of hypothesis testing, with an excellent fit to the data.

The bifactor model suggests that a general factor (problematic internet use) explains most of the variance in the PIUQ-SF-9 scores, while the three specific dimensions have distinct but smaller participation in the variance. In the original version of the instrument (12, 18) the most appropriate factorial structure was the three-factor model, that would be neglect, obsession, and control disorder. When evaluating the psychometric properties of PIUQ-SF-9 in samples from nine European countries, Laconi et al. (14) observed that the bifactor model with one general factor and two specific dimensions had an acceptable or good fit in eight out of nine subsamples. However, in that same study, the bifactor model with the three specific dimensions showed acceptable fit indices in six out of nine languages (Italian, German, Spanish, Turkish, English, and Greek). All items loaded significantly on the general factor. Item 6 (concealing the time spent online) showed the highest load in the general factor (0.87), although there was non-significant loading on the control disorder specific dimension. Item 9 (people complaining about too much time online) also loaded only in the general factor (0.70), but not on the neglect specific dimension. This was also observed in the nine subsamples evaluated in the study by Laconi et al. (14), and we may hypothesize that these behaviors are more frequent when a pattern of problematic use is already established, and when one's perception of problematic use is higher.

The internal consistency of the Brazilian version of PIUQ-SF-9 demonstrated good levels of homogeneity, as demonstrated by the analysis of both the α and the ωH indices, which is in line with previous international validating studies of the questionnaire (14, 35). It is worth mentioning that, in bifactor models, the ωH for the specific dimensions represent the reliability of a subscale score after controlling for the variance due to the general factor, explaining why these values are much smaller than the ωH value for the general factor (30).

Test–retest reliability was considered to be moderate, not differing much from other recent validation studies (21). It is possible that methodological aspects may have influenced the stability of the measure, like the time interval between the test and the retest, and also the possible different contexts in which participants responded to the instrument for the first time (during the academic year or during holidays, for example). On the other hand, we can also raise the hypothesis that problematic internet use may present variations in its natural course, either in the intensity of symptoms or in its recovery, as a chronic disorder with spontaneous remission and recurrences (20). It is also worth mentioning that both the test and the retest were carried out before the COVID-19 pandemic, which greatly interfered with the use of the internet.

Although there are dozens of instruments developed to assess problematic internet use (36), none of them is considered to be the gold standard, which makes it difficult to assess the PIUQ-SF-9's criterion validity. MIMIC analyses used to assess the construct validity was based on findings in the literature showing that problematic use is related to more time spent on the internet (not for studies or work), is more prevalent in young adults than in older age groups, and it is also often associated with psychiatric comorbidities (especially depression). All the associations and their degrees were consistent with previous studies (13, 37, 38). The greatest magnitudes were found in the positive effect of self-perception of problematic internet use on the PIUQ-SF-9's general factor (0.48) and control disorder (0.51). Interestingly, self-perception of problematic use showed a non-significant effect on the obsession dimension, perhaps because it is the most subjective dimension of the scale. Taken together, these results also reinforce the usefulness of a bifactor model.

The validation process of an instrument needs to be understood within the context in which it was used and, in this sense, it may have some limitations about the findings of this study. The first is that the cross-sectional design of the study doesn't permit to make causal inferences, and the terms “predictors” and “effect” related to the MIMIC model are only statistical predictors and effects, not real causal prediction. The second is that the study sample was selected in a non-probabilistic way, which may limit the external validity of our findings. In a continental and culturally diverse country as Brazil, it is possible that not all regions of the country have been equally represented in the study population, despite the efforts of researchers to seek this representation when recruiting the sample. We observed that both test and retest participants had a high level of education, considerably higher than the average Brazilian population (39). Although internet use in Brazil has been shown to be associated with higher levels of education (6), convenience sampling may have also influenced this finding.

In summary, based on the process of the adaptation of the PIUQ-SF-9 into Brazilian Portuguese and the validation evidence examined, the PIUQ-SF-9 seems to be a valid and reliable instrument to be used in future studies on problematic internet use in Brazil. The availability of a culturally validated instrument with sound psychometric properties will allow us to estimate and monitor the risk of problematic internet use in our population, to examine the effectiveness of prevention and treatment protocols, and also to compare these data with findings from other countries. Due to its brevity, the PIUQ-SF-9 can easily be included in research protocols without increasing significantly the completion time. This can also increase participants' compliance, especially those with more severe patterns of problematic internet use. In order to increase the PIUQ-SF-9 evidence of validation, future research should explore the instrument's measurement invariance, its performance in clinical samples and populations of different stages of development (e.g., teenagers), and also its possible gender differences in problematic internet usage (40).

## Data Availability Statement

The datasets presented in this study can be found in online repositories. The names of the repository/repositories and accession number(s) can be found in the article/Supplementary Material.

## Ethics Statement

The studies involving human participants were reviewed and approved by Research Ethics Committee of the Hospital de Clínicas de Porto Alegre (Protocol Number 89702318.2.0000.5327). The patients/participants provided their written informed consent to participate in this study.

## Author Contributions

DS, WLM, SL, KK-C, ZD, OK, IP, and SH contributed to the conception and design of the study. DS, VA, CP, and PL organized the database. DS, WLM, MY, and OK performed the statistical analysis. DS and MY wrote the first draft of the manuscript. SL, KK-C, PL, OK, ZD, and SH wrote sections of the manuscript. All authors contributed to manuscript revision, read, and approved the submitted version.

## Conflict of Interest

ELTE Eötvös Loránd University receives funding from the Szerencsejáték Ltd to maintain a telephone helpline service for problematic gambling. ZD has also been involved in research on responsible gambling funded by Szerencsejáték Ltd and the Gambling Supervision Board and provided educational materials for the Szerencsejáték Ltd's responsible gambling program. The University of Gibraltar receives funding from the Gibraltar Gambling Care Foundation. However, this funding aren't related to this study and the funding institution had no role in the study design or the collection, analysis, and interpretation of the data, writing the manuscript, or the decision to submit the paper for publication. The remaining authors declare that the research was conducted in the absence of any commercial or financial relationships that could be construed as a potential conflict of interest.
