# Supplementary material for: Psychometric Properties of the Nine-Item Problematic Internet Use Questionnaire in a Brazilian General Population Sample
Source: Front Psychiatry. 2021 May 12;12:660186. doi: 10.3389/fpsyt.2021.660186 (PMC8149803; doi:10.3389/fpsyt.2021.660186)
Supplement: Supplementary file 1 [file Table_1.DOCX]

**Appendix A. Questionário de Uso Problemático de Internet – 9 itens***

(Spritzer et al. 2021)

As perguntas abaixo se referem ao seu uso de internet de uma maneira geral (não para trabalho ou estudos). Ao responder cada pergunta, marque a opção que melhor descreve como você tem se sentido e se comportado nos últimos 6 meses, de acordo com a seguinte escala:

| 1  Nunca | 2  Raramente | 3  Algumas vezes | 4  Frequentemente | 5  Sempre/quase sempre |
| --- | --- | --- | --- | --- |

|  | 1 | 2 | 3 | 4 | 5 |
| --- | --- | --- | --- | --- | --- |
| Com que frequência você deixa de realizar tarefas domésticas para ficar mais tempo online? |  |  |  |  |  |
| Com que frequência você sente que deveria diminuir a quantidade de tempo que você passa online? |  |  |  |  |  |
| Com que frequência você fica online quando deveria estar dormindo? |  |  |  |  |  |
| Com que frequência você tem vontade de diminuir a quantidade de tempo que você passa online, mas não consegue? |  |  |  |  |  |
| Com que frequência você se sente tenso, irritado ou estressado se você não pode usar a internet pelo tempo que você gostaria no dia? |  |  |  |  |  |
| Com que frequência você tenta esconder a quantidade de tempo que você passa online? |  |  |  |  |  |
| Com que frequência você se sente tenso, irritado ou estressado se você não pode usar a internet por vários dias seguidos? |  |  |  |  |  |
| Com que frequência você se sente deprimido, mal-humorado ou nervoso quando você não está na internet e esses sentimentos passam assim que você se conecta novamente? |  |  |  |  |  |
| Com que frequência as pessoas ao seu redor reclamam do tempo que você passa online? |  |  |  |  |  |

* No previous authorization is required to use the "Questionário de Uso Problemático de Internet - 9 itens". We just require that proper credit be given to its authors and the present article be cited as a reference.
