# Supplementary material for: Psychometric Properties of the Nine-Item Problematic Internet Use Questionnaire in a Brazilian General Population Sample
Source: Front Psychiatry. 2021 May 12;12:660186. doi: 10.3389/fpsyt.2021.660186 (PMC8149803; doi:10.3389/fpsyt.2021.660186)
Supplement: Supplementary file 2 [file Table_2.DOCX]

**Appendix B. Problematic Internet Use Questionnaire – Short Form - 9 items**

(Koronczai et al. 2011)

The following questions refer to your Internet use, in a general way (not for work nor studies). As you answer each question, check the box that best describes how you have felt and conducted yourself over the past 6 months, according to the following scale: ​

| 1  Never | 2  Rarely | 3  Sometimes | 4  Often | 5  Always /almost always |
| --- | --- | --- | --- | --- |

|  | 1 | 2 | 3 | 4 | 5 |
| --- | --- | --- | --- | --- | --- |
| How often do you neglect household chores to spend more time online? ​ |  |  |  |  |  |
| How often do you feel that you should decrease the amount of time spent online? ​ |  |  |  |  |  |
| How often do you spend time online when you’d rather sleep? ​ |  |  |  |  |  |
| How often do you wish to decrease the amount of time spent online but you do not succeed? ​ |  |  |  |  |  |
| How often do you feel tense, irritated, or stressed if you cannot use the Internet for as long as you want to? ​ |  |  |  |  |  |
| How often do you try to conceal the amount of time spent online? ​ |  |  |  |  |  |
| How often do you feel tense, irritated, or stressed if you cannot use the Internet for several days? ​ |  |  |  |  |  |
| How often does it happen to you that you feel depressed, moody, or nervous when you are not on the Internet and these feelings stop once you are back online? ​ |  |  |  |  |  |
| How often do people in your life complain about spending too much time online? ​ |  |  |  |  |  |
