# Supplementary material for: Psychometric Properties of the Nine-Item Problematic Internet Use Questionnaire in a Brazilian General Population Sample
Source: Front Psychiatry. 2021 May 12;12:660186. doi: 10.3389/fpsyt.2021.660186 (PMC8149803; doi:10.3389/fpsyt.2021.660186)
Supplement: Supplementary file 3 [file Table_3.DOCX]

**Appendix C. Normative Values Based on Mean Scores of Subscales**

| Percentile | GF | Neg | Obs | CD |
| --- | --- | --- | --- | --- |
| 10% | 1,22 | 1,33 | 1,00 | 1,00 |
| 20% | 1,44 | 1,67 | 1,00 | 1,33 |
| 30% | 1,67 | 1,67 | 1,33 | 1,67 |
| 40% | 1,89 | 2,00 | 1,67 | 2,00 |
| 50% | 2,11 | 2,33 | 1,67 | 2,33 |
| 60% | 2,33 | 2,33 | 2,00 | 2,33 |
| 70% | 2,56 | 2,67 | 2,33 | 2,67 |
| 80% | 2,78 | 3,00 | 2,67 | 3,00 |
| 90% | 3,22 | 3,33 | 3,33 | 3,67 |

GF, General Factor; Neg, Neglect; Obs, Obsession; CD, Control Disorder
